# Why put all your eggs in one basket? Evolutionary perspectives on the origins of monogenic reproduction

**DOI:** 10.1038/s41437-023-00632-7

**Published:** 2023-06-16

**Authors:** Robert B. Baird, Andrew J. Mongue, Laura Ross

**Affiliations:** 1 Institute of Ecology and Evolution, University of Edinburgh, Edinburgh, EH9 3JT UK; 2 Department of Entomology and Nematology, University of Florida, Gainesville, Florida 32611 USA

**Keywords:** Evolution, Genetics, Ecology

## Abstract

Sexual reproduction is ubiquitous in eukaryotes, but the mechanisms by which sex is determined are diverse and undergo rapid turnovers in short evolutionary timescales. Usually, an embryo’s sex is fated at the moment of fertilisation, but in rare instances it is the maternal genotype that determines the offspring’s sex. These systems are often characterised by mothers producing single-sex broods, a phenomenon known as monogeny. Monogenic reproduction is well documented in Hymenoptera (ants, bees and wasps), where it is associated with a eusocial lifestyle. However, it is also known to occur in three families in Diptera (true flies): Sciaridae, Cecidomyiidae and Calliphoridae. Here we review current knowledge of monogenic reproduction in these dipteran clades. We discuss how this strange reproductive strategy might evolve, and we consider the potential contributions of inbreeding, sex ratio distorters, and polygenic control of the sex ratio. Finally, we provide suggestions on future work to elucidate the origins of this unusual reproductive strategy. We propose that studying these systems will contribute to our understanding of the evolution and turnover of sex determination systems.

## Introduction

Sexual reproduction is an ancient feature among eukaryotes and in many cases involves the evolution of two separate sexes: male and female. However, although the downstream gene networks controlling the differential development of the sexes tend to be relatively conserved (Salz 2011), the upstream mechanisms of sex determination are strikingly diverse and undergo significant transitions over relatively short timescales (Bachtrog et al. 2014). Among animals, most species exhibit genetic sex determination (GSD). GSD mechanisms themselves are diverse and include the male (XY/X0) and female (ZW/Z0) heterogametic, haplodiploid as well as the hermaphroditic systems that are common throughout the tree of life. In no clade is the diversity of sex determination mechanisms more obvious than in insects, where virtually every known type of sex determination exists (Sanchez 2008; Bachtrog et al. 2014; Blackmon et al. 2017).

In most systems with GSD, sex is determined by the genotype of the offspring. For example, in X0 systems and some XY systems, including *Drosophila*, the primary signal for sex determination is the X chromosome dose (Erickson and Quintero 2007). In other XY systems, such as the housefly *Musca domestica*, it is a Y-linked male-determining factor (Hediger et al. 1998). Likewise, in Z0 or ZW systems, such as moths and butterflies (Lepidoptera), sex can be determined by Z dosage (Sahara et al. 2012) or W-linked female-determining factors (Kiuchi et al. 2014). In haplodiploid sex determination systems, haploid males and diploid females develop from unfertilised and fertilised eggs, respectively (Evans 2004). In rare instances, however, an individual’s sex can be fated by the genotype of the mother instead of that of the offspring. This phenomenon, sometimes referred to as ‘sex predetermination’ (Ullerich 1980; Nigro et al. 2007) is often characterised by females producing single-sex broods. In other words, mothers are genetically predetermined to produce a particular sex ratio, and those that produce predominantly or exclusively male offspring are genotypically distinct from those that produce predominantly or exclusively female offspring. When mothers specialise in producing only one sex, it is referred to as monogenic reproduction (Metz 1938).

Monogeny, or split-sex ratios, occurs in over 20 different eusocial genera of Hymenoptera (Meunier et al. 2008), where colonies specialise in producing either male (drones) or female reproductives (queens), although both types of colonies also produce female workers. In the ant species *Formica glacialis*, monogeny is associated with a 5.5 Mb supergene that occurs exclusively in females in a heterozygous state, causing them to produce queens (Lagunas-Robles et al. 2021). Some parasitoid wasps are also known to produce single-sex broods, and this is controlled by multiple factors including host size, temperature, local mate competition, diet and maternal genotype (for a review see King 1987). Outside of Hymenoptera, monogeny is reported in three dipteran families, all of which have a solitary lifestyle and which include pests of agricultural significance (Hall et al. 2012; Shin et al. 2013; Scott et al. 2014, Fig. 1A): the dark-winged fungus gnats (Sciaridae, henceforth ‘fungus gnats’), the gall midges (Cecidomyiidae) and the blowflies (Calliphoridae).

**Fig. 1:**
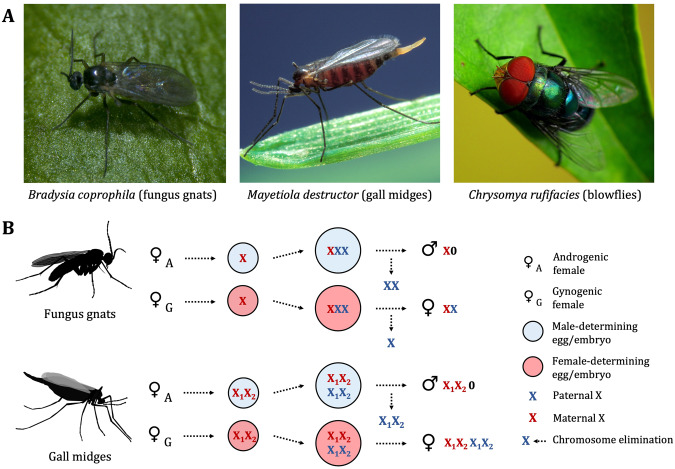
Monogenic reproduction in Diptera. **A** Monogeny is reported in three families of Diptera: dark-winged fungus gnats (Sciaridae), gall midges (Cecidomyiidae), and blowflies (Calliphoridae). **B** Sex determination via postzygotic chromosome elimination in fungus gnats and gall midges. The mechanism of sex determination is similar in the two families, with the subtle distinction that zygotes in fungus gnats are triploid because sperm provide two X chromosomes. One extra chromosome is eliminated from embryos as a result. Photo credit: J Niland (*C. rufifacies*); S Bauer (*M. destructor*); RB Baird (*B. coprophila*).

Fungus gnats and gall midges are both members of the superfamily Sciaroidea and are themselves large families comprising over 5000 and 2000 described species respectively (Skuhrava 2006; Shin et al. 2013). Several fungus gnat species are synanthropic and are receptive to being cultured in laboratory conditions, and as such they are the most well-studied of the three clades in terms of their genetics and sex determination (for reviews see Sánchez 2010; Gerbi 2022). Fungus gnats and gall midges have a non-Mendelian inheritance system called paternal genome elimination (PGE), a phenomenon in which the paternally-inherited autosome and sex chromosome copies are lost during the meiotic divisions of the spermatocytes and are therefore not transmitted to a male’s offspring. They also determine sex via elimination of paternal X chromosomes during early embryogenesis, though the exact molecular mechanism by which this is governed remains unknown (Stuart and Hatchett 1991; Gerbi 2022). The monogenic blowflies are, in comparison, relatively understudied and very little is presently known about their sex determination (Scott et al. 2014). Moreover, the evolutionary origins of monogenic reproduction, and the precise mechanisms by which it occurs, remain unknown in all three clades. Elucidating how this strange reproductive strategy evolves may help our understanding of how and why some systems depart from classical Mendelian inheritance.

Here we review current knowledge of monogenic reproduction in the three dipteran clades in which it is known to exist. We choose to focus explicitly on these dipteran examples, as there is already an extensive literature on the phenomenon in the Hymenoptera (Herre 1985; Greeff 1996; Meunier et al. 2008). In the literature, female fungus gnats are referred to as gynogenic if they are female producers and androgenic if they are male producers (e.g. Sánchez 2010), whereas in gall midges and blowflies they are referred to as thelygenic and arrhenogenic females, respectively (e.g. Stuart and Hatchett 1991; Scott et al. 2014). For the purpose of simplicity, for all clades we will refer to female producers as gynogenic and male producers as androgenic. After reviewing the three clades, we discuss evolutionary forces that may drive transitions to monogeny.

## Fungus gnats (SCIARIDAE)

Fungus gnats have been studied since the 1920s (Metz 1925) and their complicated system of chromosome inheritance has long been appreciated. The majority of our knowledge comes from the study of the closely related species *Bradysia coprophila*, *B. impatiens* and *B. ocellaris*, though the more distant *Trichosia splendens* has also been studied and shares many of the unusual features found in *Bradysia* (Metz 1938; Carson 1946; Amabis et al. 1979; Fuge 1994). Their chromosome cycle involves three rounds of PGE, one of which occurs during embryonic cleavage divisions 7–9 and is the moment when sex is determined. Unusually, fungus gnat zygotes begin with three X chromosomes. This is a result of asymmetric segregation of the X chromosome in male meiosis II, which gives rise to XX sperm. As a result, either one or two paternally-derived X chromosomes are eliminated from the embryo, which initiates female (XX) or male (X0) development, respectively (Fig. 1B). The chromosomes bound for elimination fail to divide at anaphase and are left behind on the metaphase plate, though the precise mechanism by which this elimination is controlled is unknown (DuBois 1933).

Not all fungus gnat species are strictly monogenic. Some are described as digenic, meaning they produce mixed-sex broods, though progeny sex ratios are highly variable (Davidheiser 1947). These variable sex ratios are also temperature dependent, with a higher proportion of females being produced at higher temperatures. This is caused by an increase in female production at the expense of male production rather than higher mortality in male embryos (Nigro et al. 2007; Farsani et al. 2013). The temperature-sensitive period of development appears to be the mid-pupal to early-adult stages (Nigro et al. 2007), when oogenesis takes place (Berry 1941).

Digenic and monogenic fungus gnats determine sex via the same mechanism of paternal X elimination during embryogenesis (DuBois 1933; Perondini et al. 1986). Monogeny is also known to be associated with chromosomal inversions in *B. coprophila* and *B. impatiens*, and in both cases these inversions are X-linked. The affected chromosome is termed the X´ (prime) chromosome. Gynogenic females are heterozygous for this chromosome and transmit it to half of their offspring (Carson 1946; Crouse 1960, 1979). The inverted portion is paracentric, and is terminal in *B. impatiens* (Carson 1946) but in *B. coprophila* it lies in the middle of the left arm of the X; it is not known whether the inversions occurred prior to divergence between the two species or evolved independently. We recently found that the X´ in *B. coprophila* appears to carry a supergene of multiple, linked inversions that span ~55 Mb of the ~67 Mb chromosome and emerged <0.5 mya (Baird et al. 2022). Presumably, the X´ contains the locus or loci that results in one paternal X being retained in the embryos of X´X females, while those of XX females eliminate both paternal X chromosomes. Maternally-produced factors are postulated to mediate X elimination by recognising an X-linked element. This ‘controlling element’ (CE) has been localised to the short right arm of the X. Rather than a control site, the CE likely acts as a recognition site for X elimination: if translocated to an autosome, the receiving autosome is instead eliminated (Crouse 1960; 1979; de Saint Phalle and Sullivan 1996). The X´ inversions in these monogenic species prevent homologous pairing and recombination with the X chromosome, preserving the maternal factors responsible for X elimination (Metz 1938).

Monogenic and digenic reproductive strategies are reported to exist within several distinct fungus gnat genera, including *Bradysia*, *Lycoriella*, *Scatopsciara*, and *Corynoptera*. Some species, such as *B. ocellaris*, are reported to have both monogenic and digenic strains (Metz 1938, Supplementary Table 1). Furthermore, we recently found that the X´ chromosome of *B. coprophila* evolved as recently as <0.5 mya (Baird et al. 2022). Taken together, these observations indicate that monogeny may have evolved repeatedly within the fungus gnat family, which suggests that this reproductive strategy may confer some selective advantage. The factors that drive turnover between digenic and monogenic reproduction will be discussed below.

## Gall midges (CECIDOMYIIDAE)

Gall midges represent one of the most species-diverse families of flies, comprising over 5000 known species (Skuhrava 2006; Dorchin et al. 2019). They are relatively closely related to fungus gnats; both are thought to have originated from the more primitive family Mycetophilidae. Gall midges exhibit a range of unusual reproductive strategies. Some genera of the more early-diverging subfamilies Heteropezinae and Lestemiinae reproduce via larval or pupal pedogenesis (a type of cyclic parthenogenesis involving asexual reproduction by immature insects), though the majority of species reproduce sexually (White 1973). While the chromosome cycles in Mycetophilidae are orthodox, those of gall midges, like in fungus gnats, involve several rounds of maternally-controlled elimination of paternal chromosomes, including loss of the paternal homologs during spermatogenesis (White 1973). *Mayetiola destructor* is the most well-characterised cecidomyiid in terms of sex determination and chromosome inheritance (Stuart and Hatchett 1991). This species has two pairs of nonhomologous sex chromosomes, X_1_ and X_2_. All zygotes begin with the same chromosome constitution, X_1M_X_2M_X_1P_X_2P_ (X_M_ = maternally-derived; X_P_ = paternally-derived), following the fusion of X_1_X_2_-bearing eggs and X_1_X_2_-bearing sperm. Like in fungus gnats, sex is determined when a round of X chromosome elimination occurs during the early cleavage divisions (Fig. 1B). Embryos that lose the paternal set develop into males (X_1M_X_2M_00); those that retain their X chromosomes develop into females (X_1M_X_2M_X_1P_X_2P_). X elimination is presumably governed by maternally-deposited factors in the early embryo, although this has not been confirmed.

Although gall midge species from various genera have been documented as strictly monogenic, some species exhibit both monogenic and digenic reproductive strategies. (Supplementary Table 2). The model species *M. destructor* is one example of a species with both monogenic and digenic females. The mechanism of sex determination via X elimination is the same in *M. destructor* embryos regardless of whether the broods are single- or mixed-sex. In this species, gynogenic and androgenic females are distinguished by an autosomal inversion, for which gynogenic females are heterozygous. Because the inversion is present only in female producers, it is only ever found in females and is inherited by half of the offspring in a regular Mendelian fashion such that an equal ratio of gynogenic and androgenic females are produced (Stuart and Hatchett 1991). Presumably, the inversion contains one or more loci that repress X elimination in the embryo, while acting to suppress recombination and prevent the transfer of the locus or loci to the homologous autosome. The inversion spans ~2 Mb, corresponding to around 1.3% of the haploid genome (Benatti et al. 2010; Vellacott-Ford 2020). Some populations of *M. destructor* also have a second, ~3 Mb nonoverlapping inversion present only when the first inversion is also present in *cis*. No recombination has been observed between the two inversions, suggesting that the second may have been selected for because it further suppresses recombination along the chromosome (Benatti et al. 2010).

Despite being separated by over 147 million years of evolution and several intermediate families (Ševčík et al. 2016; Hodson et al. 2022), Sciaridae and Cecidomyiidae share many features including monogeny, a near-identical chromosome cycle with PGE, as well the presence of germline-restricted chromosomes (GRCs) which are eliminated from somatic cells in early development. It is therefore tempting to speculate on a common origin for some of the features of these two clades. It was recently discovered that the GRCs in *B. coprophila* share little homology with the core chromosomes of their host species, but rather are closer in sequence similarity to the core genome of *M. destructor*, likely being acquired by fungus gnats from gall midges via introgression between 114 and 50 mya (Hodson et al. 2022). If PGE did not evolve independently in the two lineages then it must have either been lost in the intermediate families, or otherwise perhaps also transferred through introgression. Moreover, if monogenic reproduction has evolved repeatedly in the fungus gnats then it may have also done so in the gall midges, though phylogenetic information on gall midges with different reproductive strategies is lacking.

## Calliphoridae (BLOWFLIES)

Among the blowflies, only two species, *Chrysomya rufifacies* and *C. albiceps*, have been described as monogenic (Wilton 1954; Ullerich 1958); other members of this genus have male heterogamety with differentiated X and Y sex chromosomes. In the genera *Lucilia* and *Cochliomyia*, which lack monogeny, sex is controlled via a male-determining Y factor that initiates autoregulatory splicing of the sex-determination cascade gene *transformer* (Concha and Scott 2009; Li et al. 2013). In the non-monogenic *Chrysomya* species *C. chlorophyga*, aberrant X0 and XXY embryos develop into females and males, respectively, which suggests that the ancestral mechanism for monogenic species involves a Y-linked male determining locus (Ullerich 1976). In contrast, monogenic *Chrysomya* reportedly have undifferentiated sex chromosomes (Ullerich 1975). Andere et al. (2020) performed coverage-based assignment of ~3.3 and 1.5 Mb worth of sequence to putative X and Y chromosomes, respectively, suggesting that there may be some sex-linked regions, but this requires further work to fully resolve. The mechanism by which sex is determined in monogenic blowflies remains unknown, but it is likely to be fundamentally different from fungus gnats and gall midges since chromosome transmission behaviour appears to be regular, with no reports of PGE or X elimination. Furthermore, fungus gnats and gall midges belong to the lower Diptera (Nematocera) superfamily Sciariodea and are therefore relatively closely related (Ševčík et al. 2016). Blowflies, on the other hand, are higher dipterans (Brachycera), which diverged from lower dipterans ~200 mya (Wiegmann et al. 2011). A common origin for monogenic reproduction between the three families can thus be confidently ruled out.

Gynogenic female blowflies produce androgenic and gynogenic female offspring at a 1:1 ratio, and therefore the gynogenic females are thought to be heterozygous for a dominant allele that is inherited in a regular Mendelian fashion and pre-determines female sex in their offspring (Ullerich 1996, Fig. 2). Transplantation of ovaries and pole cells between androgenic and gynogenic females revealed that this sex-determining factor is synthesised by the germline during early oogenesis and maternally deposited in the embryo (Ullerich 1980, 1984). Studies of the inheritance of various genetic markers showed incomplete linkage between the markers and the locus that pre-determines offspring sex (Ullerich 1996), and translocation experiments revealed that it is situated on the proximal half of the long arm of chromosome 5 (Ullerich 1975). However, it has not yet been demonstrated whether there is recombination suppression between chromosome regions of gynogenic females, like there is in *B. coprophila* and *M. destructor*, and cytogenetic analysis of the polytene chromosomes have not revealed any obvious chromosomal rearrangements (Puchalla 1994). The genomes of male and androgenic and gynogenic female *C. rufifacies* were recently published (Andere et al. 2020), though their poor contiguity (>100,000 contigs per genome) makes identifying the control locus in gynogenic females challenging, particularly if sex is under the control of a small genomic region.

**Fig. 2:**
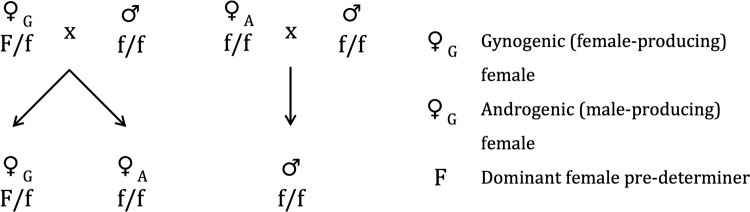
Gynogenic blowflies are heterozygous for a dominant factor that causes them to produce female offspring. The factor shows Mendelian inheritance, i.e. it is always inherited by half the daughters. If androgenic females were heterozygous for a dominant male-determining factor, then the factor would pass through both sexes and females would not always produce androgenic and gynogenic daughters in equal proportions.

Previously, *transformer* (*tra*) has been proposed as a candidate for the sex-determining locus in monogenic *Chrysomya* (Scott et al. 2014). *Tra* is one of a set of genes in a conserved cascade that regulates sexual development in many insects (Hopkins and Kopp 2021). Interestingly, mutant housefly, *M. domestica*, females that lack *tra* default to male production (Hediger et al. 2010). If such a transition were favoured in populations of *Chrysomya*, the resulting male-biased population sex ratio might subsequently drive the evolution of maternally-acting factors that cause female production. Identification and characterisation of *tra* in monogenic *Chrysomya* and its potential role in sex determination remains to be investigated.

## How does monogeny evolve?

Why would mothers evolve to produce single-sex broods? Attempts to explain the split sex ratios found in Hymenoptera focus mainly on kin selection, (Meunier et al. 2008; Kobayashi et al. 2013), inbreeding, and local mate competition in the context of their eusocial lifestyle (Herre 1985; Greeff 1996; Schrempf et al. 2006). In social Hymenoptera, diploid females and haploid males are produced from fertilised and unfertilised eggs, respectively. This results in workers being more closely related to sisters than to brothers, though this depends on the number of queens in the colony: workers are more closely related to one another when there are fewer queens. Kin selection predicts that workers should favour the production of females where relatedness between workers is higher, and that when relatedness is lower, more males should be produced (Boomsma and Grafen 1990). Empirical results indeed show this to be the case (Meunier et al. 2008). Previous hypotheses for the evolution of monogeny in fungus gnats and gall midges have focused on inbreeding depression and conflict over the sex ratio, respectively (Haig 1993; Tabadkani et al. 2011). We elaborate on both below, and suggest an additional hypothesis based on multi-locus control of the sex ratio that has some support from previous studies of fungus gnats and gall midges.

### Inbreeding depression

Inbreeding, e.g. mating between siblings, is widespread in natural populations of animals (Lacy 1993). Inbreeding increases homozygosity which leads to phenotypic expression of deleterious recessive mutations and resulting fitness costs (Pusey and Wolf 1996; Crnokrak and Roff 1999; Keller 2002; Mongue et al. 2016). These effects have been found to result in the evolution of diverse inbreeding avoidance mechanisms such as dispersal (Szulkin and Sheldon 2008), intentional avoidance of kin (Facon et al. 2006) and polyandry (Firman and Simmons 2008).

Monogenic reproduction has been suggested as an alternative mechanism for inbreeding avoidance (Tabadkani et al. 2011; Andere et al. 2020). A consequence of monogeny is that mating between siblings is impossible because the progeny in any one brood are of the same sex. Offspring must therefore disperse in order to mate, which will at worst result in mating with half-siblings. Simulations suggest that monogeny provides a potentially effective route to inbreeding avoidance, particularly when populations are small (Tabadkani et al. 2011), though empirical evidence to support this is lacking.

### Sex ratio selection

Fisherian sex ratio theory posits that in a large, randomly mating population, frequency-dependent selection should result in a 1:1 male:female sex ratio (Fisher 1930). However, there are circumstances where biased sex ratios can be advantageous. Probably the most frequent scenario is local mate competition (LMC), which occurs when matings frequently occur between close relatives. Under the most extreme scenario where matings occur between full-sibs, extremely female-biased sex ratios are selected for. Generally, in species with frequent LMC, mothers are able to facultatively adjust their brood sex ratio relative to the expected degree of sibmating. However, experimental evolution studies in mites show that this sex ratio strategy can be genetically determined (Macke et al. 2014). Although LMC can drive the evolution of a female-producing strategy, it is difficult to envisage how a male-producing strategy can evolve, because in the absence of LMC mothers should produce equal, not male-biased sex ratios (West 2009).

Sex ratio distortions can also arise where a particular sex, chromosome, or endosymbiont favours the production of one sex over another (Sandler et al. 1959; Jones 1991; Hurst 1993). One scenario that can lead to biased sex ratios is sex chromosome meiotic drive, where the transmission of one sex chromosome is favoured over the other (Jaenike 2001; Lindholm et al. 2016). For example, Gershenson (1928) showed that an X-linked factor in male *D. obscura* kills Y-bearing sperm, resulting in a female-biased sex ratio. In some cases, autosomal segregation distorters are also known to cause sex ratio distortions (Larracuente and Presgraves 2012). Meiotic drive and segregation distortion are well-studied in *Drosophila* (Courret et al. 2019) and also occurs in other Diptera (Wood and Newton 1991; Fry and Wilkinson 2004). Unlike with female-bias caused by LMC, significant departures from an even population sex ratio that occur due to drive may provide a selective advantage to parents who are able to specialise in producing the rarer sex, such that the population sex ratio returns to 1:1. Haig (1993) suggested that a driving X chromosome arising in a fungus gnat ancestor initiated the evolution of its strange chromosome cycle. Following the female-biased sex ratio that results from X-drive, mothers began converting XX daughters into X0 sons by eliminating a paternal X in the embryo, and ensuing conflict over the sex ratio ended with mothers specialising in the production of a particular sex.

The presence of supernumerary chromosomes can also cause departures from an even sex ratio. For example, B chromosomes found in many species favour the production of individuals in which they are carried, and are able to bias the sex ratio through association with nuclear-transmitted segregation distorters (Jones and Rees 1982). B chromosomes drive male-biased sex ratios in a variety of systems including fairy shrimp *Branchipus schaeferi* (Beladjal et al. 2002), the teleost fish *Astyanax scabripinnis* (Vicente et al. 1996) and the wasp *Nasonia vitripennis* (Nur et al. 1988). Supernumerary chromosomes that somewhat resemble B chromosomes (GRCs, or germline-restricted chromosomes) are found in gall midges and fungus gnats. The GRCs are eliminated from somatic cells early in development of both sexes, but are retained in the ovaries or testes (Hodson and Ross 2021). Haig (1993) noted that, because GRCs in *B. coprophila* are disproportionately transmitted by males, they should favour male-biased sex ratios. The GRCs would have thus favoured the conversion of XX daughters into sons by mothers, which may have spurred the evolution of the X´ chromosome that suppressed the actions of the GRCs. In contrast, GRCs in gall midges are exclusively transmitted through females and should therefore favour female production. The function of GRCs in fungus gnats and gall midges, and whether they have any effect on sex determination, remains to be explored.

Maternally-inherited microorganisms present another route by which conflict over the sex ratio can arise. *Wolbachia* are common reproductive parasites, and the feminisation, parthenogenesis, male-killing and cytoplasmic incompatibility that they induce is well documented in insects (Werren et al. 2008). *Wolbachia* are found in blowflies (Mingchay et al. 2014; Xu et al. 2022), including the monogenic *C. albiceps* (Şaki̇ and Şi̇mşek 2014), and have been suggested as a mechanism for biological pest control for members of this family (Caleffe et al. 2019). A *Rickettsia* genome was sequenced along with the recently-sequenced *B. coprophila* genome (Urban et al. 2021). *Rickettsia* are a group of proteobacterial endosymbionts related to *Wolbachia* that are also known to exhibit meiotic drive behaviour (Werren et al. 1994; Lawson et al. 2001; Giorgini et al. 2010), providing another potential mechanism that may have favoured the evolution of these monogenic systems.

### Polygenic control of the sex ratio

Within the digenic (mixed-sex brood producers) fungus gnats and gall midges, significant departures from a 1:1 progeny sex ratio are the norm (Davidheiser 1947; Mcclay 1996; Nigro et al. 2007), and some species are described as exhibiting mixed (both monogenic and digenic) strategies (McCarthy 1945; Steffan 1974; Stuart and Hatchett 1991; Rocha and Perondini 2000, Supplementary Tables 1 and 2). Sex ratios in these families appear to exist along a continuum, with extreme sex ratios (i.e. monogeny) fixed in some species (in blowflies variable sex ratios have not been reported). Even strictly monogenic females of *B. coprophila* do occasionally produce ‘exceptional’ offspring of the wrong sex, showing that the capacity for producing both sexes is retained in females of monogenic species (Metz and Schmuck 1929). Metz (1938) originally suggested that in *B. coprophila* the X and X´ are distinguished not by a single allele but rather a series of alleles of varying potency, and that the difference between monogenic and digenic species is the ‘strength’ of the X´ chromosome.

**Fig. 3:**
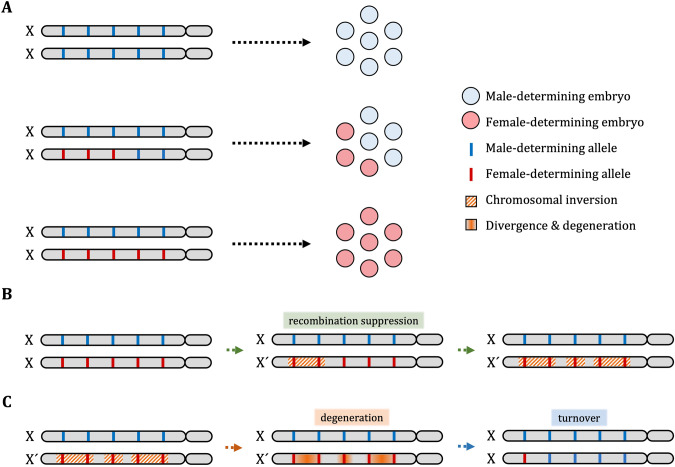
A model for the evolution of monogenic from digenic reproduction in fungus gnats (also applies to gall midges, though inversions in the gall midge *M. destructor* are autosomal). **A** Different combinations of X-linked alleles are responsible for variable sex ratios among digenic females. ‘Male-determining’ alleles should result in X elimination and ‘female-determining’ alleles should result in X retention. **B** The ‘trapping’ of female-determining alleles through recombination suppression (e.g. inversions) leads to the fixation of monogenic reproduction in a population. Alternatively, inversions may occur first, onto which female-determining alleles migrate. **C** Non-recombining X´ chromosomes degenerate and their carriers suffer reduced fitness. Individuals with X chromosomes bearing female-determining alleles reinvade the population, spurring turnover in reproductive strategy.

It is now known that the X´ in *B. coprophila* is distinguished from its X homologue by a large region of recombination suppression composed of inversions (Crouse 1979; Baird et al. 2022). Although all females of digenic species like *B. ocellaris* are XX, there must be something that genotypically distinguishes females that produce male-biased broods from those that produce female-biased broods. Davidheiser (1947) reported that the sex ratio in this species is heritable: the female offspring of female-biased broods and male-biased broods also produce female-biased and male-biased broods, respectively. In the same study, it was shown that it was possible to artificially select for predominantly male production from predominantly female production, and vice versa, in only a handful of generations. These observations of (i) continuous variation of this phenotype, (ii) inheritance of the sex ratio and (iii) rapid artificial selection provide a strong indication that the sex ratio has an additive genetic component. It follows that in this system, particular combinations of alleles at multiple loci may determine the amount of maternally deposited factors in the oocytes, which then affects the proportion of embryos that develop as male or female. Recombination between these loci in digenic lineages produces the different sex ratios observed (Fig. 3A); their fixation in some lineages leads to monogenic females with single-sex broods (Fig. 3B). Under this scenario, the term ‘monogenic’ refers to lineages in which the production of single-sex broods has become the dominant strategy in a population. This may occur via recombination suppression via inversions around the dominant female-determining alleles. Alternatively, inversions may occur first and then alleles that modify the sex ratio may migrate to inverted regions. Females without inversions should then evolve more male-biased production as an evolutionary response, with the expectation being that the genotype heterozygous for the inversions is maintained at 50% in the population by frequency-dependent selection.

The distribution of monogenic, digenic and mixed reproductive strategies across fungus gnats suggests multiple evolutionary origins for monogeny within the family, or perhaps frequent reversions to digeny (Supplementary Table 1). This is further supported by the finding that the X´ chromosome in *B. coprophila* emerged only <0.5mya. Turnover between monogenic and digenic reproduction may therefore be common. Since the non-recombining region degenerates over time (Baird et al. 2022), decreased fitness in X´X females may present opportunities for females that produce mixed-sex progenies to invade, resulting in reversions to digeny (Fig. 3C).

Why would monogeny evolve under this scenario? Skewed sex ratios in digenic species may be evidence of divergent selection acting on the sex ratio. If control of the sex ratio is indeed polygenic, an initial distorting driver may not be required. Sex determination systems that are under polygenic control are thought to be inherently unstable, because if one sex-determining locus provides a fitness benefit over others then that locus should eventually fix as the sole sex-determiner (Rice 1986). Instability of polygenic sex determination is also thought to be exacerbated in small populations where it is more likely to produce skewed sex ratios and where rarer alleles may be lost more frequently by drift or selection (Bateman and Anholt 2017). In monogenic populations with a non-recombining X´, the trapped sex ratio alleles act as a single locus that may resolve instability in digenic populations.

## Conclusions and future perspectives

Monogenic reproduction is one of the most unusual forms of genetic sex determination and its origins remain elusive. In the blowflies, too little is currently known about sex determination in the *Chrysomya* genus to speculate on the origins of monogeny in this clade. It will be essential to first determine what distinguishes androgenic and gynogenic females genotypically in monogenic *Chrysomya*, as well as to characterise the sex determination systems and sex ratios in non-monogenic members of the genus.

As for fungus gnats and gall midges, the relationship between digenic and monogenic reproduction is unclear and the question of multiple independent origins for monogeny remains open. To answer these questions it will be essential to sequence and compare the genomes of different monogenic species within the families, especially species that are more distantly related to the *Bradysia* models studied thus far. In particular, the development of chromosome-level assemblies will aid in comparative genomics and identification of inversions associated with monogeny. The closely related *B. coprophila* and *B. impatiens* both harbour X´ chromosomes that are slightly different in structure. Their relationship is unknown, but it could provide an indication as to whether and how monogeny evolves repeatedly. It will also be necessary to uncover the molecular control of the sex ratio in monogenic and digenic species, and to determine the role of the GRCs, if any, in sex determination. Lab colonies of *B. coprophila* have been maintained since the 1920s (Moses and Metz 1928), and more recently, colonies of other species including digenic *Lycoriella ingenua* have been established (RB Baird and L Ross, unpublished studies). The genome of the model *B. coprophila* is now available (Urban et al. 2021), and site-specific insertions of DNA as well as piggyBac-mediated transformation techniques have recently been developed for this species (Yamamoto et al. 2015; Yamamoto and Gerbi 2022); as such there are increasingly available opportunities to understand more about this system.

Moreover, further work is required to determine the selective forces that drive transitions to monogeny. Inbreeding may appear an unlikely explanation, since inbreeding is widespread (Lacy 1993), whereas monogeny is not. As for resolution of sex ratio distortions, evolving monogeny from the ancestral sex determination system may be more difficult than simply evolving suppressors of drive (Atlan et al. 2003). However, intrinsic properties of fungus gnats and gall midges may mean that they are more amenable to evolving monogeny. Since non-monogenic members of these families already have variable progeny sex ratios, the transition to extreme sex ratios may be a relatively straightforward solution to suppress drive or resolve inbreeding depression. Furthermore, sex ratios in digenic fungus gnats may be face instability due to the temperature effect on their progeny sex ratios: environmental sex determination is thought to be unstable in the face of environmental perturbations, which destabilise sex ratios, giving rise to GSD (Van Dooren and Leimar 2003). In contrast, blowflies may represent a more major transition from an XY system to monogeny, which might require a stronger selective pressure to evolve.

Also striking are some of the features that are shared by these systems. For example, monogeny seems to be associated with chromosomal inversions. The study of these systems may therefore broaden our understanding of how inversion-based supergenes are associated with the evolution of complex traits (Schwander et al. 2014). Furthermore, so far it always appears to be the female producers that are the heterogametic morphs that possess these inversions. If it were instead male producers that carried heterozygous inversions, then they would need to pass through males which might present opportunities for genetic conflicts between the sexes.

More generally, these systems offer unique opportunities to study the evolution of sex determination systems and sex ratios. The consensus in the literature is that the optimal mean sex ratios that individuals produce should be broods of equal numbers of males and females (Frank 1990). Producing anything other than 1:1 progeny sex ratios is rare, and understanding why this is the case requires studying systems that deviate from the norm. Together, exploration of the sex determination systems of these peculiar flies may help inform us about how some of the most fundamental mechanisms in evolution – of sex determination systems and sex ratios – evolve.

## Supplementary information

Supplementary material
