# Supplementary material for: Why put all your eggs in one basket? Evolutionary perspectives on the origins of monogenic reproduction
Source: Heredity (Edinb). 2023 Jun 16;131(2):87–95. doi: 10.1038/s41437-023-00632-7 (PMC10382564; doi:10.1038/s41437-023-00632-7)
Supplement: Supplementary file 1 — Supplementary material [file 41437_2023_632_MOESM1_ESM.docx]

**Supplementary material for:**

Why put all your eggs in one basket? Evolutionary perspectives on the origins of monogenic reproduction

Robert B. Baird^1^*, Andrew J. Mongue^2^ and Laura Ross^1^.

1. Institute of Ecology and Evolution, University of Edinburgh, Edinburgh, EH9 3JT, UK

2. Department of Entomology and Nematology, University of Florida, Gainesville, Florida 32611

*Corresponding author

Email: robert.baird@ed.ac.uk

**Supplementary table 1.** Reported reproductive strategies across dark-winged fungus gnats (Sciaridae). *Bradysia*, *Phytosciara*, *Scatopsciara* and *Ctenosciara* belong to the monophyletic group Megalosphyinae; *Lycoriella* belong to the non-monophyletic group Pseudolycoriella; *Corynoptera* belong to either Pseudolycoriella or the non-monophyletic Cratyninae [(Shin et al. 2013)](https://www.zotero.org/google-docs/?MQEztS). The positions of the *Cosmosciara*, *Hyperlasion* and *Rhynchosciara* genera are unclear.

| **Genus** | **Species** | **Reported strategy** | **References** |
| --- | --- | --- | --- |
| *Bradysia* | *coprophila / tilicola* | Monogenic | [(Metz 1938)](https://www.zotero.org/google-docs/?1ZZGeK) |
|  | *impatiens / difformis* | Monogenic |  |
|  | *ocellaris / tritici* | Mixed |  |
|  | *varians* | Monogenic |  |
|  | *reynoldsi* | Digenic |  |
|  | *prolifica* | Digenic |  |
|  | *fenestralis* | Mixed | [(McCarthy 1945a)](https://www.zotero.org/google-docs/?0s0f1B) |
|  | *spatitergum* | Monogenic | [(Steffan 1974)](https://www.zotero.org/google-docs/?c1O6Wu) |
|  | *molokaiensis* | Monogenic |  |
|  | *bishopi* | Digenic |  |
|  | *matrogrossensis* | Mixed | [(Rocha and Perondini 2000)](https://www.zotero.org/google-docs/?d7jLcz) |
|  | *paupera* | Monogenic | [(Liu 2007)](https://www.zotero.org/google-docs/?V3N4jm) |
|  | *odoriphaga* | Mixed | [(Cheng et al. 2017)](https://www.zotero.org/google-docs/?tv5DNN) |
| *Phytosciara* | *vulcanata* | Digenic | [(Steffan 1974)](https://www.zotero.org/google-docs/?eJxfEN) |
| *Scatopsciara* | *nacta* | Monogenic | [(McCarthy 1945a)](https://www.zotero.org/google-docs/?uhYZ4H) |
|  | *nigrita* | Digenic | [(Steffan 1974)](https://www.zotero.org/google-docs/?oAxEDS) |
|  | *cunicularius* | Mixed |  |
| *Ctenosciara* | *hawaiiensis* | Monogenic |  |
| *Lycoriella* | *similans* | Mixed | [(Metz 1938)](https://www.zotero.org/google-docs/?oyCuz7) |
|  | *ingenua / mali / pauciseta* | Digenic |  |
|  | *agraria* | Digenic | [(McCarthy 1945b)](https://www.zotero.org/google-docs/?QeudFw) |
|  | *solispina* | Monogenic | [(Steffan 1974)](https://www.zotero.org/google-docs/?6pQmha) |
|  | *hoyti* | Digenic |  |
|  | *aurpilia* | Mixed | [(Binns 1980)](https://www.zotero.org/google-docs/?E0kyZq) |
| *Corynoptera* | *subtrivialis* | Monogenic | [(Metz 1938)](https://www.zotero.org/google-docs/?GIFgFt) |
|  | *brevipalpis* | Digenic | [(Steffan 1974)](https://www.zotero.org/google-docs/?rVCVkf) |
| *Cosmosciara* | *perniciosa* | Digenic |  |
| *Rhynchosciara* | *hollaenderi* | Monogenic | [(Mattingly and Dumont 1971)](https://www.zotero.org/google-docs/?byADPO) |
|  | *americana / angelae* | Monogenic | [(Lara et al. 1965)](https://www.zotero.org/google-docs/?6joOSK) |
| *Hyperlasion* | *wasmanni* | Mixed | [(Mohrig et al. 2019)](https://www.zotero.org/google-docs/?RCtTCq) |

**Supplementary table 2.** Reported reproductive strategies across gall midges (Cecdiomyiidae).

| **Genus** | **Species** | **Reported strategy** | **References** |
| --- | --- | --- | --- |
| *Mayetiola* | *destructor* | Mixed | [(Stuart and Hatchett 1991)](https://www.zotero.org/google-docs/?vR523t) |
| *Rabdophaga* | *heterobia* | Monogenic | [(Barnes 1931)](https://www.zotero.org/google-docs/?rWovuY) |
|  | *saliciperda* | Monogenic | [(Kraczkiewicz 1966)](https://www.zotero.org/google-docs/?yyiwcp) |
|  | *salicisbatatas / batatas* | Monogenic | [(Geyer-Duszyńska 1961)](https://www.zotero.org/google-docs/?NWqD0w) |
| *Dasineura* | *folliculi* | Monogenic | [(Dorchin et al. 2007)](https://www.zotero.org/google-docs/?DzzoJX) |
|  | *carbonaria* | Monogenic |  |
| *Solidago* | *gigantea* | Mixed |  |
| *Izeniola* | *obesula* | Monogenic | [(Dorchin and Freidberg 2004)](https://www.zotero.org/google-docs/?R8PtSH) |
| *Orseolia* | *Oryzivora* | Monogenic | [(Omoloye 2006)](https://www.zotero.org/google-docs/?QqGWvR) |
| *Aphidoletes* | *aphidimyza* | Monogenic | [(Tabadkani et al. 2012)](https://www.zotero.org/google-docs/?amS30m) |
| *Sitodiplosis* | *mosellana* | Monogenic | [(Smith et al. 2004)](https://www.zotero.org/google-docs/?6StX4R) |
| *Cystiphora* | *sonchi* | Mixed | [(Mcclay 1996)](https://www.zotero.org/google-docs/?Xcgvuy) |

**REFERENCES**

[Barnes HF. 1931. The sex ratio at the time of emergence and the occurrence of unisexual families in the gall midges (Cecidomyidae, diptera). *J. Genet.* 24:225–234.](https://www.zotero.org/google-docs/?V9IPfB)

[Binns ES. 1980. Field and laboratory observations on the substrates of the mushroom fungus gnat *Lycoriella auripila* (Diptera: Sciaridae). *Ann. Appl. Biol.* 96:143–152.](https://www.zotero.org/google-docs/?V9IPfB)

[Cheng J, Su Q, Jiao X, Shi C, Yang Y, Han H, Xie W, Guo Z, Wu Q, Xu B, et al. 2017. Effects of Heat Shock on the *Bradysia odoriphaga* (Diptera: Sciaridae). *J. Econ. Entomol.* 110:1630–1638.](https://www.zotero.org/google-docs/?V9IPfB)

[Dorchin N, Clarkin CE, Scott ER, Luongo MP, Abrahamson WG. 2007. Taxonomy, Life History, and Population Sex Ratios of North American *Dasineura* (Diptera: Cecidomyiidae) on Goldenrods (Asteraceae). *Ann. Entomol. Soc. Am.* 100:539–548.](https://www.zotero.org/google-docs/?V9IPfB)

[Dorchin N, Freidberg A. 2004. Sex ratio in relation to season and host plant quality in a monogenous stem-galling midge (Diptera : Cecidomyiidae). *Ecol. Entomol.* 29:677–684.](https://www.zotero.org/google-docs/?V9IPfB)

[Geyer-Duszyńska I. 1961. Spindle disappearance and chromosome behavior after partial-embryo irradiation in Cecidomyiidae (Diptera). *Chromosoma* 12:233–247.](https://www.zotero.org/google-docs/?V9IPfB)

[Kraczkiewicz Z. 1966. Premiers stades de l’oogenèse de *Rhabdophaga saliciperda* (Cecidomyiidae, Diptera). *Chromosoma* 18:208–229.](https://www.zotero.org/google-docs/?V9IPfB)

[Lara FJS, Tamaki H, Pavan C. 1965. Laboratory Culture of *Rhynchosciara angelae*. *Am. Nat.* 99:189–191.](https://www.zotero.org/google-docs/?V9IPfB)

[Liu Y. 2007. Chemoecological Studies on the Reproductive Behaviors of the Darkwinged Fungus Gnat, *Bradysia paupera* (Diptera: Sciaridae, doctoral thesis).](https://www.zotero.org/google-docs/?V9IPfB)

[Mattingly E, Dumont JN. 1971. Early spermatogenesis in *Rhynchosciara*. *In Vitro* 6:286–299.](https://www.zotero.org/google-docs/?V9IPfB)

[McCarthy MD. 1945a. Chromosome Studies on Eight Species of *Sciara* (Diptera) with Special Reference to Chromosome Changes of Evolutionary Significance. II (Continued). *Am. Nat.* 79:228–245.](https://www.zotero.org/google-docs/?V9IPfB)

[McCarthy MD. 1945b. Chromosome Studies on Eight Species of *Sciara* (Diptera) with Special Reference to Chromosome Changes of Evolutionary Significance. *Am. Nat.* 79:104–121.](https://www.zotero.org/google-docs/?V9IPfB)

[Mcclay AS. 1996. Unisexual broods in the gall midge *Cystiphora sonchi* (Bremi) (Diptera: Cecidomyiidae). *Can. Entomol.* 128:775–776.](https://www.zotero.org/google-docs/?V9IPfB)

[Metz CW. 1938. Chromosome behavior, inheritance and sex determination in *Sciara*. *Am. Nat.* 72:485–520.](https://www.zotero.org/google-docs/?V9IPfB)

[Mohrig W, Kauschke E, Broadley A. 2019. Revision of black fungus gnat species (Diptera, Sciaridae) described from the Hawaiian Islands by DE Hardy and WA Steffan, and a contribution to the knowledge of the sciarid fauna of the Galápagos Islands. *Zootaxa* 4590:401–439.](https://www.zotero.org/google-docs/?V9IPfB)

[Omoloye AA. 2006. Sex ratio bias in the F1 adult progeny of African rice gall midge, *Orseolia oryzivora* H. and G. (Dipt., Cecidomyiidae). *J. Appl. Entomol.* 130:349–355.](https://www.zotero.org/google-docs/?V9IPfB)

[Rocha LS, Perondini ALP. 2000. Analysis of the sex ratio in *Bradysia matogrossensis* (Diptera, Sciaridae). *Genet. Mol. Biol.* 23:97–103.](https://www.zotero.org/google-docs/?V9IPfB)

[Shin S, Jung S, Menzel F, Heller K, Lee H, Lee S. 2013. Molecular phylogeny of black fungus gnats (Diptera: Sciaroidea: Sciaridae) and the evolution of larval habitats. *Mol. Phylogenet. Evol.* 66:833–846.](https://www.zotero.org/google-docs/?V9IPfB)

[Smith MAH, Wise IL, Lamb RJ. 2004. Sex ratios of *Sitodiplosis mosellana* (Diptera: Cecidomyiidae): implications for pest management in wheat (Poaceae). *Bull. Entomol. Res.* 94:569–575.](https://www.zotero.org/google-docs/?V9IPfB)

[Steffan WA. 1974. Laboratory studies and ecological notes on Hawaiian Sciaridae (Diptera). *Pac. Insects* 16:41–50.](https://www.zotero.org/google-docs/?V9IPfB)

[Stuart JJ, Hatchett JH. 1991. Genetics of Sex Determination in the Hessian Fly, *Mayetiola destructor*. *J. Hered.* 82:43–52.](https://www.zotero.org/google-docs/?V9IPfB)

[Tabadkani SM, Allahyari H, Farhoudi F, Rahimi-Alangi V, Ramin S. 2012. Effect of male densities on sex ratio variations of the predatory gall midge, *Aphidoletes aphidimyza* (Diptera: Cecidomyiidae).](https://www.zotero.org/google-docs/?V9IPfB)
